# Uncovering Shortcomings in Advertising Strategies for Over-the-Counter Minoxidil Products on Amazon

**DOI:** 10.7759/cureus.58656

**Published:** 2024-04-20

**Authors:** Kritin K Verma, Becky Joseph, Helen Chen, Daniel P Friedmann, Michelle Tarbox

**Affiliations:** 1 School of Medicine, Texas Tech University Health Sciences Center, Lubbock, USA; 2 Dermatology, Texas Tech University Health Sciences Center, Lubbock, USA; 3 Dermatology and Cosmetic Surgery, Westlake Dermatology & Cosmetic Surgery, Austin, USA

**Keywords:** amazon, adverse effects transparency, advertising strategies, minoxidil, over-the-counter (otc) market

## Abstract

Introduction

The over-the-counter (OTC) market for hair loss products, particularly those containing minoxidil, has significantly expanded due to the increased prevalence of hair loss. Minoxidil, a vasodilator medication, is known for its potential to stimulate hair growth. However, the rise in OTC formulations has led to misleading advertising and marketing, with some companies exaggerating the benefits of their products while minimizing potential adverse effects.

Methods

A Google Boolean Search was conducted to identify OTC minoxidil products. The topmost non-sponsored search engine result page was used for analysis. Products not containing any dosage of minoxidil were excluded, resulting in nine products. These were individually searched on Amazon and eight were analyzed for any addressed safety information and adverse effects profile.

Results

The analysis revealed that only two out of eight products (25%) reported safety information, and none of the products (0%) reported any adverse effects. Significant observations were found surrounding the transparency and accuracy of the advertising and marketing of these products. Many companies made bold claims about their products without providing supporting scientific evidence or studies. Furthermore, many of these OTC hair loss brands did not adequately mention and explain the adverse effects of the product.

Conclusions

The study highlights the need for greater transparency in the marketing of OTC minoxidil products. Companies should provide clear and accessible information about the safety and potential adverse effects of their products. This will empower consumers to make informed decisions and foster trust between the industry and the consumer. Furthermore, the authenticity and accuracy of marketing images should be ensured to avoid giving false hopes to consumers.

## Introduction

The over-the-counter (OTC) market for hair loss products has significantly expanded in response to the increased prevalence of hair loss in individuals. Although there are many options available, minoxidil is one of the major OTC hair loss medications on the market. 

Minoxidil, a vasodilator medication, has gained attention for its potential to stimulate hair growth by extending the anagen (growth) phase of the hair cycle and increasing the size of hair follicles [1]. Some data suggests that the stimulatory action of minoxidil on hair growth may be due to its sulfated metabolite, minoxidil sulfate, activating potassium channels [1]. However, exploring this mechanism of action has proved to be difficult, suggesting that further research is needed, especially in confirming evidence that potassium adenosine triphosphate (KATP) channels are expressed in the hair follicle [1]. Minoxidil-based OTC hair loss treatments have grown in popularity over the years. There are many different companies that sell these products, and some of the most widely-used formulations are topicals, such as solutions and foams applied to affected areas [2]. The rise in OTC formulations of the drug has its advantages and disadvantages. On one side, buying these products over the counter offers consumers convenience and affordability that prescription drugs may not have. However, it also exposes individuals to misleading advertising and marketing. Furthermore, it is concerning that certain companies selling these OTC hair loss products make it seem that these products can provide a complete solution to the issue, while they lack or make information difficult to find regarding the safety of the product as well as adverse effects. 

The primary objective of this paper is to evaluate OTC minoxidil products, specifically on Amazon, as it is one of the most frequently used online websites, and investigate whether the companies address safety and adverse effects. The aim of this paper is to investigate OTC minoxidil products and the advertising strategies that companies may employ to boost sales that may result in a misinformed treatment decision and false hope.

## Materials and methods

A Google Boolean search was conducted using the following: “list AND hair AND loss AND growth AND serums OR solution.” Out of the 255,000,000 results generated online, the topmost non-sponsored search engine result page, also known as the most frequent landing site (MFLS) for a specific search, was utilized in our analysis. Using 10 MFLS online articles and magazines [3-12], products advertised as shampoo and/or conditioners intended for eyebrow use were all excluded. A total of 58 unique products were reported, all of which were unisex/female-indicated serums or solutions for scalp use only. Only nine products had minoxidil dosing mentioned on the label and were included; all other products were excluded from the study. From there, products were individually searched on Amazon, as our objective was to analyze the most popular online website, and eight were found and analyzed for any addressed safety information and adverse effects profile.

## Results

The ingredient lists of standard OTC hair loss serums and solutions from the MFLS yielded eight products catered to hair growth that used minoxidil 2% or 5% on Amazon. Table 1 summarizes a list of which products were analyzed and what their relative concentrations were [13-17].

**Table 1:** Safety Information and Adverse Effects Profile List of Over-the-Counter Serums & Solutions Categorized by Minoxidil Concentrations.

Minoxidil Dosage	Over-the-Counter Serums & Solution Names	Safety Information Mentioned (Yes/No)?	Adverse Effects Profile Listed (Yes/No)?
Minoxidil 2%	Keranique Hair Regrowth Treatment [13]	Yes	No
	Nioxin Hair Regrowth Treatment for Women [14]	No	No
	hers Hair Regrowth Treatment [15]	No	No
Minoxidil 5%	Keeps Extra Strength Minoxidil for Men [16]	Yes	No
	Virtue Hair Growth Treatment 1 Month Kit [17]	No	No
	TOMUM 5% Minoxidil Foam for Men and Women [18]	No	No
	Lilivera 5% Minoxidil Spray [19]	No	No
	Women's Rogaine 5% Minoxidil Foam [20]	No	No

Two products (25%) reported safety information regarding the use of the products, and none of the products (0%) reported any adverse effects.

## Discussion

When analyzing the eight OTC minoxidil products from the search, significant observations were found surrounding the advertising and marketing of the products, including elements of transparency and accuracy in marketing materials. 

Transparency is required in building trust with consumers so that they can make informed decisions regarding their hair loss treatment plan. Without medical professionals guiding the consumer in drug usage, all the information regarding the drug must be easily accessible and clear to the consumer. The lack of transparency is apparent when examining products on websites like Amazon. For instance, TOMUM boldly claims that "no topical hair loss treatment works faster," and this claim could significantly influence a customer's decision [18]. However, there is a paucity of scientific evidence that has focused on speed and efficacy to back this claim regarding OTC minoxidil treatment. Leaving out this crucial piece of information may leave consumers wondering about the accuracy of such claims, and this underlines how necessary it is to have transparency through supporting studies and references.

In addition, Keranique states that 100% of users regrew their hair while using their serum, while 98% agreed it provided thicker, fuller hair [13]. These statements are perfect to grab a consumer's attention. However, they raise the same concerns that TOMUM brings. While Keranique mentions that these claims are backed by a study, accessing the study, as well as the methodology behind the study, seems to be challenging. Regarding transparency, direct access or links to the studies must be provided in order to assess the accuracy of the claims. Furthermore, while Keranique presents these statistics about their products, when searching their website for a study, it was found that the website has a generic minoxidil study instead of one that directly assesses the effectiveness of the brand product. The linked study states, “[I]n clinical studies of mostly white women aged 18-45 years with mild to moderate degrees of hair loss, the following response to minoxidil topical solution 2% was reported: 19% of women reported moderate hair regrowth after using minoxidil topical solution 2% for 8 months (19% had moderate regrowth; 40% had minimal regrowth)" [21]. This does not align with the statements that the brand advertises, and the study’s link to the Keranique brand remains unclear. 

Transparency also becomes a concern for OTC hair loss products when looking at the ingredients used in the products and how they are presented to consumers. While many companies place their focus on minoxidil being an FDA-approved OTC drug for hair loss, the problem occurs when non-FDA-approved ingredients are added to the formulations. For example, the brand Virtue states it is the “most scientifically advanced hair growth line ever made.” Under this claim, they list many ingredients, such as “Larchwood extract, probiotic ferment, and biomimetic signal peptides" [22]. Minoxidil is not mentioned in this list at all. These ingredients do not have FDA approval and can give the misleading impression that the entire formulation is backed by substantiated scientific evidence and overestimate the product's efficacy. Furthermore, it seems that many of these OTC hair loss brands do not adequately mention and explain the adverse effects of the product. Minoxidil is an effective vasodilator that has been demonstrated to increase hair growth and help reduce hair loss in people with androgenetic alopecia (AGA) [23]. However, topical minoxidil can result in minor scalp dryness and irritation, which can cause itching and discomfort [23]. Additionally, it may also develop adverse effects, such as allergic contact dermatitis, a skin irritation brought on by an allergy to minoxidil that manifests as scalp redness, itching, and occasionally blistering [23].

Furthermore, minoxidil synchronization of the hair cycle can temporarily exacerbate hair loss by causing the shedding of hairs already in the telogen (resting) phase of the hair cycle [24]. Another adverse reaction to minoxidil can also lead to a condition known as hypertrichosis [25]. As mentioned, many adverse effects may occur due to minoxidil, but these were challenging or nonexistent when trying to locate them in product descriptions and marketing materials. OTC Minoxidil product inserts must mention all important and relevant adverse effects, or else the onus of litigious claims in the court of law will prove costly. Companies should adhere to science and make information regarding safety and adverse effects available on websites like Amazon. There is a lack of clear information and accessibility, which makes it difficult for consumers to make informed decisions and raises concerns about the brand's commitment to consumer safety and well-being. In order to be sure that there is ethical use of marketing for OTC hair loss products, transparency must be presented regarding ingredient information and adverse effects associated with certain products. This empowers consumers to make decisions and fosters trust between the industry and the consumer. 

When examining OTC minoxidil products, another issue is the authenticity and accuracy of marketing images. Some images display before and afters regarding certain products that give patients unrealistic expectations of their hair growth [18, 26]. The before and after images from Lilivera, for example, contain the same exact photo positioning, lighting, and quality. However, the only difference is that the after photo has more hair [18]. Although speculatory, producing an image that is so close to that quality is nearly impossible, unless it is altered by Photoshop. Additionally, as seen in Tomum’s product, the middle image with the before and after pictures is deceiving, as there is a noticeable swirl or circular pattern in the before image, however, it is completely lacking in the after picture [18]. Our suspicions note that it may be harder to photoshop something of that quality, and, therefore, it is more visible to see that something like that was changed. Images like these alter the credibility of such companies, give patients false hopes in regard to the potential outcomes of OTC minoxidil products, and may lead to the consumer being disappointed when their own results do not align. Alarmingly, this could harm consumers' emotional and financial well-being and may have the opposite effect on hair growth in certain cases [23]. To maintain integrity and trust within the industry of OTC hair loss products, it is important that there is a strong commitment to accuracy with marketing materials, especially through visual evidence. 

In the OTC minoxidil product market, it is evident that brands use different tactics to capture consumers' attention and evoke emotions, which may sway their decisions. When searching for minoxidil products on Amazon, it was found that some products had labels such as “Amazon Choice” or “Ending 50% off sale” in a specific time frame. While these strategies aim to attract and entice consumers into buying the products, consumers should not use this as the primary approach when considering what OTC hair loss products to use. Though labels like “Amazon Choice” are determined by ratings, price, popularity, product availability, and fast delivery and should still be considered in consumer decision-making, the main factor that should be considered is the brand's reliability based on scientific evidence [27]. Though these medications are not prescription drugs, they still significantly impact a user's health and well-being. Taking this into account, consumers should be careful when buying products in this market; they should analyze which products are credible and what may best suit their needs based on the potential benefits and the adverse effects. In addition to these tactics, emotional persuasion through images and statements seems to be another way companies sway individuals to purchase their products. Keranique highlights a consumer statement that says, “I had a big giant bald spot right in the front” which easily captures the attention of an individual suffering from the same issue [13]. Though these statements may be based on factual experience from a consumer, the disparity lies in how they are worded as well as their size and visibility, while vital details about the product’s formula, scientific evidence, and even adverse effects are hard to locate or comparatively small in size. Therefore, it seems to show that brands are more interested in luring in consumers based on emotional responses than helping them make an informed decision. 

Though this study was written in order to gain insight into advertising and marketing practices in regard to OTC minoxidil products, there are limitations that must be acknowledged. First, the method used to search for products was a Google Boolean search derived from the most commonly searched magazines and websites. Therefore, certain products may have been omitted from our results. Additionally, each product may have multiple sellers on Amazon. However, in our analysis, we only used the most popular listing. 

## Conclusions

The evaluation of over-the-counter (OTC) minoxidil treatments raises serious concerns about transparency, accuracy, and customer trust. Many OTC minoxidil treatments' advertising materials are unclear and do not give scientific proof to back up their efficacy claims, hurting consumer confidence. Furthermore, the emphasis on non-FDA-approved chemicals over minoxidil, an FDA-approved hair loss medicine, misleads customers about the product's safety and effectiveness. Inadequate communication of potential minoxidil side effects, such as contact dermatitis and hair loss, further restricts informed decision-making. Marketing strategies such as distorted before-and-after photographs and emotional persuasion can lead to unreasonable expectations and have a negative influence on consumers' emotional and financial well-being. To build industry trust and empower customers, better transparency, precise ingredient portrayal, explicit side effect disclosure, and ethical marketing methods are required. Furthermore, patients should attempt to resort to a licensed dermatologist for their hair growth needs instead of making decisions based on influencers and/or social media marketing. When selecting OTC hair loss solutions, consumers should prioritize credible information and product compatibility, keeping in mind their substantial health and well-being.
